# Sensor-based outcomes to monitor everyday life motor activities of children and adolescents with neuromotor impairments: A survey with health professionals

**DOI:** 10.3389/fresc.2022.865701

**Published:** 2022-10-12

**Authors:** Fabian Marcel Rast, Rob Labruyère

**Affiliations:** ^1^ Swiss Children’s Rehab, University Children’s Hospital Zurich, Affoltern am Albis, Switzerland; ^2^ Children’s Research Center, University Children’s Hospital of Zurich, University of Zurich, Zurich, Switzerland; ^3^ Rehabilitation Engineering Laboratory, Department of Health Sciences and Technology, ETH Zurich, Zurich, Switzerland

**Keywords:** pediatric rehabilitation, activities of daily living, motor performance, wearable inertial sensors, algorithm development, international classification of functioning, disability and health

## Abstract

In combination with appropriate data processing algorithms, wearable inertial sensors enable the measurement of motor activities in children's and adolescents' habitual environments after rehabilitation. However, existing algorithms were predominantly designed for adult patients, and their outcomes might not be relevant for a pediatric population. In this study, we identified the needs of pediatric rehabilitation to create the basis for developing new algorithms that derive clinically relevant outcomes for children and adolescents with neuromotor impairments. We conducted an international survey with health professionals of pediatric neurorehabilitation centers, provided them a list of 34 outcome measures currently used in the literature, and asked them to rate the clinical relevance of these measures for a pediatric population. The survey was completed by 62 therapists, 16 doctors, and 9 nurses of 16 different pediatric neurorehabilitation centers from Switzerland, Germany, and Austria. They had an average work experience of 13 ± 10 years. The most relevant outcome measures were the duration of lying, sitting, and standing positions; the amount of active self-propulsion during wheeling periods; the hand use laterality; and the duration, distance, and speed of walking periods. The health profession, work experience, and workplace had a minimal impact on the priorities of health professionals. Eventually, we complemented the survey findings with the family priorities of a previous study to provide developers with the clinically most relevant outcomes to monitor everyday life motor activities of children and adolescents with neuromotor impairments.

## Introduction

In pediatric neurorehabilitation, children and adolescents with congenital and acquired illnesses and injuries of the developing brain are treated and cared for. These children and adolescents often present neurological impairments that result in difficulties in executing everyday life motor tasks, such as walking to school, grasping a glass of water, or transferring from a wheelchair to a car seat. They undergo intensive therapy programs as in- or out-patients with the emphasis on reducing these limitations and fostering their functional independence in everyday life. Here, motor assessments are essential for developing a patient-centered therapy plan, monitoring the children's progress over time, and providing families with objective information. These assessments are usually conducted at the clinic in a standardized environment. However, after discharge (in-patients) or between therapy sessions (out-patients), the children's social and environmental factors become more important. Hence, it remains unclear whether children can translate their improvements during rehabilitation into everyday life at home or school. Assessing the children's motor performance by measuring what children actually do in their habitual environment would overcome this limitation (1). Consequently, there is a need for scientifically sound tools to measure performance in children and adolescents with neuromotor impairments.

Today, motor performance is predominantly assessed with self- or proxy-report questionnaires, which are prone to recall or proxy bias (2). Activity counts derived from body-worn accelerometers have been used as an objective and unbiased alternative to assess performance. While these counts provide valid estimates of total energy expenditure (3) or non-specific hand use (4), they do not capture information about the type of performed activities (5). In contrast, the use of multiple state-of-the-art motion sensor modules in combination with appropriate data processing algorithms would allow for the determination of activity-specific outcome measures (e.g., the time a child spent in a sitting position, the child's self-selected speed of walking periods, or how often a child was grasping an object in daily life, etc.). Over the years, a large variety of algorithms deriving different aspects of everyday life motor activities of people with mobility impairments have been developed (6). However, the outcomes of these algorithms were predominantly designed for adult patient populations which triggers the question of whether these outcomes are relevant for children and adolescents with neuromotor impairments.

Besides this large variety of algorithms designed for adults, there is also a handful of algorithms available which were specifically developed for a pediatric population (7–13). These algorithms estimate the time spent in sedentary, standing, walking, running, cycling, or upper limb activities; discriminate between active and passive wheelchair mobility; calculate an index of hand use laterality; and determine gait parameters, such as stride time and length. However, even though it would be technologically feasible to measure these outcomes in children and adolescents with neuromotor impairments, the question remains whether they provide relevant information for pediatric rehabilitation.

In addition to the relevance of sensor-based outcomes, developers must also consider the number of required sensors and thus the wearability of the whole sensor setup. For example, measuring all of the abovementioned outcomes with sufficient accuracy would require the patient to wear eight sensors concurrently, which we expect would jeopardize the children's and adolescents' willingness to wear these devices in daily life. Previous studies about the usability of inertial sensors showed that sensors need to be comfortable, discreet, and unobtrusive to not affect daily behavior and to be accepted by the end-user (14–16). These studies' findings imply the need to minimize the number of body-worn sensors. Consequently, the development of new algorithms will be a trade-off between maximizing information gain and minimizing the number of sensors (17).

Therefore, developers of new algorithms that generate meaningful outcomes for children and adolescents with neurological impairments need to know the clinical needs of pediatric neurorehabilitation. With this information, developers can make decisions about the abovementioned trade-off. To determine the needs of children and families, we investigated their mobility and self-care rehabilitation goals on an activity level according to the International Classification of Functioning, Disability, and Health. The results of this study have been published elsewhere, and the five most frequent rehabilitation goals were walking short distances, transferring oneself while sitting, putting on clothes, going up and downstairs, and maintaining a sitting position (18). In the current study, we aimed to complement the families' needs with the opinion of pediatric health professionals. We conducted a survey with doctors, nurses, and therapists of pediatric neurorehabilitation centers, provided them a list of outcome measures currently used in the literature (6), and asked them to rate the clinical relevance of these measures for children and adolescents with neuromotor impairments. Eventually, we aimed to provide a priority list of sensor-based outcomes for pediatric rehabilitation.

## Materials and methods

The data collection of this study was anonymous, and we did not collect health-related data from the survey participants. This type of study does not require ethical approval in Switzerland.

### Development and description of the survey

**Figure 1:**
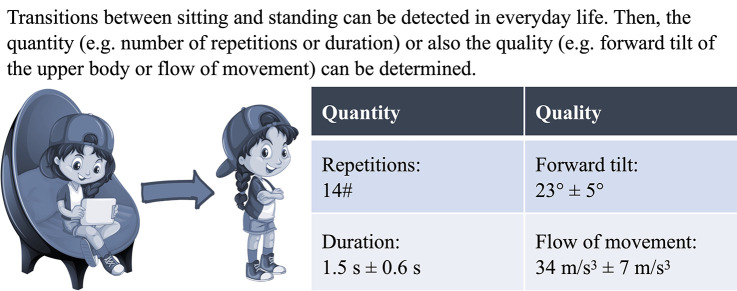
An example of the presentation of two survey items: the quantity and quality of sit-to-stand transitions. The illustration was designed using resources from freepik.com.

The survey comprised 34 items, each representing an outcome measuring the quantity or quality of a motor activity performed in daily life (Table 1). The items were derived from a systematic review providing an overview of all sensor-based outcome measures applied in people with mobility impairments (6). Related items were grouped into categories, and each category contained a brief description of the outcomes and, if applicable, a graphical visualization of a fictitious measurement. An example of such a measurement is provided in Figure 1. At the end of each category, there was an open-ended question asking about possible other relevant outcomes not covered by the survey.

**Table 1:** Description and categorization of all 34 survey items.

ID	Item	Description
Arm and hand use		Upper limb movements can be measured separately for the left and right arm. This enables the quantification of the hand use laterality, the amount of unimanual and bimanual activities, and the diversity of upper limb movements.
1	Hand use (laterality)	
2	Hand use (uni-/bimanual)	
3	Hand use (diversity)	
Joint movement		Joint angles can also be measured. This can be used to quantify the number of repetitions and the range of motion of individual joints in everyday life.
4	Shoulder abd/add	
5	Elbow flex/ex	
6	Forearm pro/sup	
7	Wrist flex/ex	
8	Finger flex/ex	
9	Knee flex/ex	
Reaching / grasping		Reaching and grasping movements can be detected and evaluated in everyday life. This allows quantifying the number of repetitions as well as the range of reaching forward and sideward relative to the trunk.
10	Reaching (repetitions)	
11	Reaching (range)	
Maintaining a body position		Lying, sitting and standing can be recognized in everyday life and the duration a child spends in these body positions is measured. Lying can be subclassified as prone, supine, and side lying, and standing as upright, bending forward, or bending sideward.
12	Lying, sitting / standing	
13	Lying (prone/supine)	
14	Standing (upright/bent)	
Changing a body position		Transitions between sitting and standing can be detected in everyday life. Then, the quantity (e.g., number of repetitions or duration) or also the quality (e.g., forward tilt of the upper body or flow of movement) can be determined.
15	Standing up (quantity)	
16	Standing up (quality)	
Walking activity		Walking can be distinguished from other activities, and the daily walking activity can be divided into individual walking bouts. Then, the duration, distance and speed of these bouts can be determined.
17	Walking (duration)	
18	Walking (distance/speed)	
Gait parameters		Walking can be segmented into gait cycles which allows quantifying gait parameters such as step length, duration of the stance phase or step symmetry.
19	Walking (gait parameters)	
Risk of falling		From walking activities, different measures can be calculated that predict a child's risk of falling.
20	Risk of falling	
Walking (turning)		Obstacles or a side road can force a change of direction during walking activities. These turns can be analyzed regarding speed, angular change, number of steps, etc.
21	Walking (turning)	
Walking (slope)		The slope of covered walking routes can be measured which allows determining whether a child can walk in steep terrain. In addition, the gait pattern can be compared between level, uphill, and downhill walking.
22	Walking (slope)	
Stair climbing		Stair climbing periods and the covered number of steps can be recorded in everyday life (quantity). Furthermore, it can be distinguished between a step-by-step and a step-over-step pattern (quality).
23	Stair climbing (quantity)	
24	Stair climbing (quality)	
Use of walking aids		The use or non-use of assistive devices can be assessed for walking and other activities. Other measures, such as weight bearing or the orientation/position of the assistive device could be determined, too.
25	Use of walking aids	
Wheelchair		Wheeling activities can be detected and subclassified as passive wheeling (being pushed by a third person or a motor) or active self-propulsion. Furthermore, the covered distance and the speed can be determined.
26	Wheeling (active/passive)	
27	Wheeling (distance/speed)	
Activities of daily living		Various other activities of daily living can be detected, and the duration or the number of repetitions of these activities can be determined. Here, the activities were grouped because the possibilities are very diverse.
28	School activities	
29	Personal hygiene	
30	Dressing	
31	Eating / drinking	
32	Household activities	
33	Sports activities	
Energy expenditure		The intensity of physical activities can be measured and divided into three levels (low, medium and high intensity). This allows determining the daily energy expenditure.
34	Energy expenditure	

flex, flexion; ex, extension; pro, pronation; sup, supination; abd, abduction; add, adduction.

The participants were asked to rate the clinical relevance of each item/outcome measure on a four-point Likert scale, including the responses (1) very relevant, (2) relevant, (3) hardly relevant, and (4) not relevant for the rehabilitation of children and adolescents. Participants also had the possibility of not answering an item. At the beginning of the questionnaire, the participants were instructed to imagine that children or adolescents would wear a sensor system in their habitual environment, and the system would be able to derive the outcome measures described in the survey. Moreover, participants were asked to rate the clinical relevance from an interdisciplinary perspective.

We created the survey with the web application Findmind (https://findmind.ch). The content of the original survey is provided in Supplementary file 1. The survey was pretested with a 28-year-old female physiotherapist, a 33-year-old male occupational therapist, and a 32-year-old female nurse to check and improve the clarity of the questions. Filling out the survey took roughly 15 min.

### Distribution of the survey

The target populations were doctors, nurses, and therapists of pediatric neurorehabilitation centers in the German-speaking part of Europe. We sent the link of the online survey to the directors of 23 pediatric neurorehabilitation centers in Switzerland, Germany, and Austria. We asked them to forward this link to their medical, nursing, and therapy staff with the request to participate. We distributed the survey in April 2018, sent a reminder in May 2018, and closed the survey in August 2018.

The survey was password-protected to avoid unauthorized participation. The data collection was anonymous to protect the privacy of the participants, which in turn made it impossible to prevent multiple participation. The data was kept confidential and was encrypted with the Secure Socket Layer protocol.

### Statistical analysis

For each item, the number of responses was counted for each response level. Then, missing values were imputed with the weighted mean of the k nearest-neighbors, with k being chosen as the number of participants. The estimates of the missing values were rounded to the nearest integer to reflect real responses. This approach was chosen because it applies to non-parametric data (19) and avoids overestimating the relevance rating of items without missing responses compared to those with missing responses because missing responses decrease the potential rank of the remaining items.

The clinical relevance of the items was investigated with the median response and the mean rank. The mean rank was determined by ranking the responses of each participant and averaging the ranks of all participants for each item. The ranks were adjusted for ties by assigning the average of the ranks that would have been assigned without ties. Eventually, the items were sorted by their clinical relevance.

The random sample of this survey was not balanced across potential confounders such as the participants' health profession, their work experience in pediatric rehabilitation, and their workplace. Therefore, we investigated the influence of these confounders on the responses of the survey. The Kruskal-Wallis test was used to elaborate if the responses of each item differed between health professions. In case of significant differences, the Tukey's range test was applied for profession-wise comparisons. The relationship between work experience and the responses of each item was determined with the Spearman rank correlation coefficient. Since the majority of participants worked at the Swiss Children's Rehab, we decided to allocate the participants' workplace into two groups consisting of participants working at the Swiss Children's Rehab and those working at other rehabilitation centers. Then, the potential bias of responses from people working at the Swiss Children's Rehab was investigated with the Wilcoxon rank sum test. The alpha level of all statistical tests was set to 0.05. It was not corrected for multiple testing to avoid overlooking potential influences of the chosen confounders on the relevance ratings. The analysis was conducted with MATLAB R2018b (The MathWorks, Inc.; Natick; USA).

## Results

**Figure 2:**
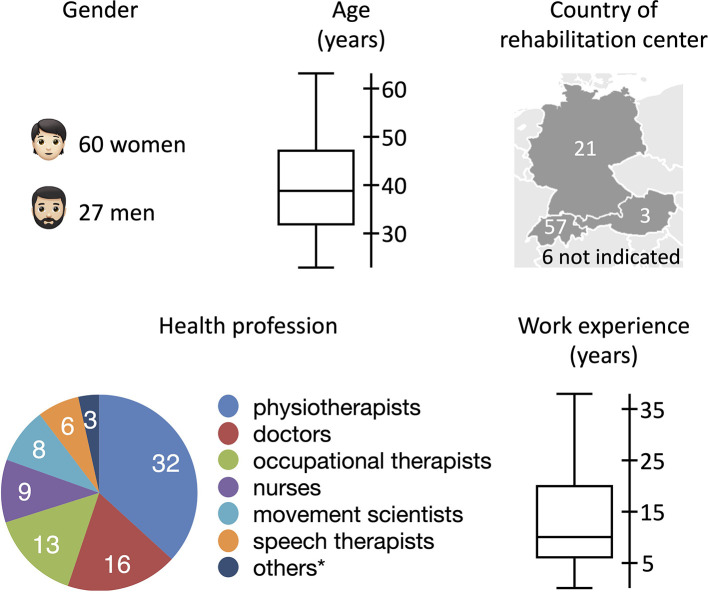
Participants' characteristics. *1 sport therapist, 1 Special Education teacher, and 1 team of doctors and therapists.

The survey was filled out by 87 health professionals from 16 different pediatric neurorehabilitation centers. Hence, 70% of the centers that we contacted participated in this study. The participants' characteristics are illustrated in Figure 2. Forty-one participants worked at the Swiss Children's Rehab, while the remaining participants worked at different rehabilitation centers distributed across the German-speaking part of Europe.

The total counts of individual responses, the median response, and the mean rank of each item are listed in Table 2. The items are sorted in ascending order by their mean rank and thus start with the most relevant item for children and adolescents with neuromotor impairments. All items received a median rating of “very relevant” or “relevant”. In total, 64 items were not answered, which leads to a rate of missing items of 2%. The open-ended questions at the end of each category were filled out in 11% of all cases. The given answers are summarized in Table 3. The most frequently mentioned outcomes not explicitly covered in the survey were the step length, the duration of different gait phases, and how fast patients can change their direction during walking activities; the posture of the trunk and pelvis while maintaining a certain body position (e.g., sitting); the amount of playing activities; and the range of shoulder flexion/extension movements.

**Table 2:** Priority list of sensor-based outcome measures including the total counts of individual responses, the median response, and the mean rank of each outcome.

ID	Item	# very relevant	# relevant	# hardly relevant	# not relevant	# missing value	median	mean rank
12	Lying, sitting / standing	64	19	3	0	1	1	12.1
26	Wheeling (active/passive)	59	22	6	0	0	1	13.1
1	Hand use (laterality)	57	28	2	0	0	1	13.5
18	Walking (distance/speed)	54	30	3	0	0	1	13.9
17	Walking (duration)	51	31	4	1	0	1	14.6
20	Risk of falling	52	23	9	0	3	1	14.7
25	Use of walking aids	54	25	6	2	0	1	14.7
2	Hand use (uni-/bimanual)	49	33	5	0	0	1	14.9
31	Eating / drinking	48	31	6	1	1	1	15.5
23	Stair climbing (quantity)	42	42	3	0	0	2	15.7
15	Standing up (quantity)	42	35	8	0	2	2	16.2
30	Dressing	42	38	4	2	1	2	16.3
19	Walking (gait parameters)	42	34	8	0	3	1.5	16.3
11	Reaching (range)	44	31	11	0	1	1	16.4
9	Knee flex/ex	39	34	10	0	4	2	16.9
16	Standing up (quality)	41	34	11	1	0	2	17.0
5	Elbow flex/ex	40	32	11	0	4	2	17.3
29	Personal hygiene	38	38	7	2	2	2	17.4
8	Finger flex/ex	35	37	10	0	5	2	17.8
33	Sports activities	29	51	5	0	2	2	18.2
3	Hand use (diversity)	35	36	14	1	1	2	18.3
27	Wheeling (distance/speed)	33	43	10	1	0	2	18.5
7	Wrist flex/ex	30	44	9	0	4	2	18.7
6	Forearm pro/sup	32	38	11	1	5	2	18.7
34	Energy expenditure	35	34	15	2	1	2	18.7
24	Stair climbing (quality)	35	31	20	1	0	2	19.2
14	Standing (upright/bent)	28	42	14	0	3	2	19.5
13	Lying (prone/supine)	31	30	22	0	4	2	19.9
10	Reaching (repetitions)	26	40	16	1	4	2	20.3
4	Shoulder abd/add	23	45	16	0	3	2	20.8
28	School activities	26	38	18	4	1	2	21.2
21	Walking (turning)	18	44	18	2	5	2	21.9
22	Walking (slope)	15	51	20	0	1	2	22.2
32	Household activities	10	46	21	7	3	2	24.7

A median response of 1 = “very relevant” and 2 = “relevant”.

flex, flexion; ex, extension; pro, pronation; sup, supination; abd, abduction; add, adduction.

**Table 3:** Answers of open-ended questions after each category.

Category	Relevant outcomes not covered in the survey (number of occurrences)
Arm and hand use	Task-specific arm and hand use (2×), efficiency of arm and hand use (1×), independence of arm and hand use (1×), overhead arm and hand use (1×), and fine hand use (1×).
Joint movement	Shoulder flex/ex (6×), shoulder rotation (2×), hip flex/ex (2×), ankle flex/ex (2×), scapula depression (1×), and thumb opposition and abd/add (1×).
Reaching / grasping	Workspace (3×), trunk and scapula stabilization (2×), reaching the mouth (1×).
Maintaining a body position	The posture of the trunk and pelvis during these body positions (8×), discriminating between different sitting positions (3×), use of assistive devices (2×), 4-point kneeling (2×), tilt angle of the backrest (1×), and kneeling (1×).
Changing a body position	Lying <−> sitting (3×), standing up without support (3×), sitting <−> 4-point kneeling (2×), 4-point kneeling <−> standing (2×), transferring in a sitting position (1×), efficiency of changing a body position (1×), and pressure ulcer prevention (1×).
Walking activity	Walking in different locations (2×), required effort while walking (1×) and backward walking (1×).
Gait parameters	Step length (21×), duration of individual gait phases such as stance and swing phase (19×), step width (5×), terrain (5×), presence of heel strike (4×), cadence (4×), joint range of motion (2×), use of walking aids (2×), type of footwear (2×), and posture of the pelvis (1×).
Risk of falling	With or without supervision or assistance (2×), and toe clearance (1×).
Walking (turning)	Turning speed (9×), number of steps (5×), with or without supervision or assistance (4×), and turning angle (1×).
Walking (slope)	–
Stair climbing	Use of hand rails (5×), speed (2×), leading leg (1×), orientation (e.g., sideward, backward) (1×), required effort while climbing stairs (1×), step height, and deceleration before the stair climbing period (1×).
Use of walking aids	Type of walking aids (4×), amount of weight bearing (2×), carrying an object (1×), and discriminating between in- and outdoors (1×).
Wheelchair	Type of propulsion (3×), passing curbs (2×), maneuvering (1×), number of strokes (1×), symmetry (1×), type of wheelchair (1×), and sitting duration in a wheelchair (1×).
Activities of daily living	Eating (4×), dressing (2×), caring for body parts (2×), drinking (1×), toileting (1×), writing (1×), independence (1×), and self-care activities (1×).
Energy expenditure	–
What's missing?	Playing (7×), crawling (2), independence (2×), communication (1×), jumping (1×), required effort (1×), fall detection (1×), and detection of involuntary movements (1×).

flex, flexion; ex, extension; abd, abduction; add, adduction.

The influences of the confounders on the relevance rating of the items are listed in Table 4 (same order of items as in Table 2). The relevance rating of five items differed between health professionals, while the post-hoc, profession-wise comparison revealed only three significant differences. Nurses rated the relevance of measuring the use of assistive devices higher than movement scientists and the duration of sports activities higher than physiotherapists. Besides, occupational therapists rated the relevance of measuring pro- and supination of the forearm higher than nurses. The relevance rating of eight items significantly depended on the work experience of the participants. Negative correlation coefficients mean that the relevance of these items was rated higher with increasing work experience. Determining the distance and speed of walking activities and the number of climbed stairs received a higher rating from the staff working at the Swiss Children's Rehab while measuring pro- and supination of the forearm and the duration of household activities were rated as more relevant from the staff of other rehabilitation centers.

**Table 4:** Test statistics regarding the influence of confounders on rating the relevance of sensor-based outcome measures.

ID	Item	Health profession		Work experience		Work place	
		χ^2^	*p*-value	*Ρ*	*p*-value	z-score	*p*-value
12	Lying, sitting / standing	4.5	0.48	0.00	0.99	−0.5	0.62
26	Wheeling (active/passive)	4.1	0.54	−0.04	0.73	−0.3	0.74
1	Hand use (laterality)	3.0	0.70	0.03	0.82	−1.5	0.13
18	Walking (distance/speed)	5.9	0.32	−0.02	0.84	**−2.8**	**0**.**00**
17	Walking (duration)	9.5	0.09	0.03	0.79	−0.5	0.62
20	Risk of falling	9.4	0.10	0.13	0.25	−1.7	0.09
25	Use of walking aids	**11**.**8**	**0**.**04**	−0.18	0.10	−0.7	0.50
2	Hand use (uni-/bimanual)	6.4	0.27	−0.17	0.11	−0.6	0.54
31	Eating / drinking	5.5	0.36	−0.08	0.44	0.7	0.51
23	Stair climbing (quantity)	9.3	0.10	−0.14	0.19	**−2**.**6**	**0**.**01**
15	Standing up (quantity)	5.5	0.36	**−0**.**23**	**0**.**03**	0.9	0.36
30	Dressing	6.4	0.27	−0.10	0.35	0.8	0.41
19	Walking (gait parameters)	2.2	0.82	−0.14	0.21	−1.1	0.28
11	Reaching (range)	8.2	0.15	0.00	0.98	−0.4	0.71
9	Knee flex/ex	2.2	0.82	−0.15	0.17	0.0	0.97
16	Standing up (quality)	7.1	0.22	−0.07	0.50	0.2	0.83
5	Elbow flex/ex	3.2	0.68	−0.21	0.05	0.4	0.70
29	Personal hygiene	2.3	0.81	−0.08	0.45	1.2	0.22
8	Finger flex/ex	1.8	0.88	−0.16	0.13	0.5	0.60
33	Sports activities	**13**.**3**	**0**.**02**	−0.15	0.16	−1.1	0.29
3	Hand use (diversity)	**12**.**6**	**0**.**03**	−0.16	0.15	1.3	0.21
27	Wheeling (distance/speed)	**13**.**6**	**0**.**02**	**−0**.**22**	**0**.**05**	0.1	0.90
7	Wrist flex/ex	8.5	0.13	−0.17	0.12	0.8	0.44
6	Forearm pro/sup	**11**.**4**	**0**.**04**	**−0**.**27**	**0**.**01**	**2**.**5**	**0**.**01**
34	Energy expenditure	8.0	0.16	0.01	0.93	−0.4	0.67
24	Stair climbing (quality)	3.2	0.67	**−0**.**27**	**0**.**01**	0.2	0.85
14	Standing (upright/bent)	6.9	0.23	**−0**.**26**	**0**.**02**	1.4	0.15
13	Lying (prone/supine)	6.2	0.29	**−0**.**24**	**0**.**03**	0.9	0.38
10	Reaching (repetitions)	9.4	0.09	**−0**.**24**	**0**.**02**	1.6	0.11
4	Shoulder abd/add	2.7	0.75	−0.14	0.19	1.0	0.32
28	School activities	8.8	0.12	**−0**.**28**	**0**.**01**	1.7	0.09
21	Walking (turning)	4.2	0.53	−0.20	0.06	0.7	0.51
22	Walking (slope)	8.2	0.15	−0.14	0.19	−0.8	0.42
32	Household activities	3.4	0.64	−0.21	0.05	**2**.**4**	**0**.**01**

Bold numbers indicate a significant influence of the confounder on the rating of the item's relevance.

flex, flexion; ex, extension; pro, pronation; sup, supination; abd, abduction; add, adduction.

## Discussion

In this survey, we investigated the opinion of health professionals on the clinical relevance of sensor-based outcomes to monitor everyday life motor activities of children and adolescents with neuromotor impairments.

On average, health professionals of pediatric neurorehabilitation centers rated all outcomes of the survey as being “relevant” or “very relevant” for children and adolescents with neuromotor impairments. None of the outcomes were classified as “hardly relevant” or “not relevant”. Still, the relevance measured with the mean rank of all responses differed between outcomes resulting in the priority list shown in Table 2. In activities with quantity- and quality-related outcomes, the quantity-related outcomes were prioritized. In particular, the number of climbed stairs was more relevant than the used stepping pattern, the number of sit-to-stand transitions received a higher rating than how these transitions were executed, and the duration and distance of walking activities were more relevant than outcomes assessing the underlying gait pattern. We have two explanations for prioritizing quantity over quality. First, motor tests conducted at the clinic can capture the quality of an activity and might also reflect how children are doing this activity in daily life. However, how often children are doing this activity in daily life can only be captured with wearable sensors. Second, a top priority of pediatric rehabilitation is to gain independence in mobility and self-care activities (20). And to be independent, the capability to do an activity seems to be more important than how these activities are executed. Hence, assessing the quantity of activities is probably a better indicator of independence than assessing their quality.

In the following sections, we discuss the findings of this survey with regard to three main categories: maintaining and changing a body position, walking and moving, and upper limb activity.

### Maintaining and changing a body position

The duration a child spends in a certain body position was the most relevant item and more important than changing between positions and assessing the quality of the posture. The opinion of health professionals coincides with the families' needs since maintaining a sitting and standing position was often set as a rehabilitation goal (18). Still, the top priority of families in this category was transferring oneself while sitting for patients using a wheelchair (18). However, this activity was not part of the current survey because it has not yet been assessed with wearable sensors. Therefore, the opinion of health professionals regarding the relevance of quantifying transfers in daily life remains unexplored.

### Walking and moving

The distinction between active and passive wheeling periods was the most relevant item in this category. This is surprising since it is a rare rehabilitation goal of children and adolescents (18) and shows the importance of complementing the families' needs with those of pediatric health professionals. Measuring the amount of active self-propulsion could be an indicator of independence or the level of physical activity in individuals using a wheelchair (21), potentially explaining the high relevance rating of this item. Besides wheeling, the distance and speed of walking activities received the highest rating in this category. The ability to cover a certain distance on foot is also the most frequently set goal in pediatric rehabilitation (18). It is important to reach destinations in daily life and could again be an indicator of independence. Moreover, walking speed is essential for children with neuromotor impairments to keep up with their peers (22). All this could explain the high priority to measure distance and speed of daily walking activities with wearable sensors. Health professionals further rated the walking duration, the risk of falling, the use of walking aids, the number of climbed stairs, and the gait pattern as being more relevant than the distance and speed of wheeling periods, the stepping pattern of stair climbing periods, the turning behavior while walking, and the slope of covered walking routes.

### Upper limb activity

The hand use laterality and the distinction between unimanual and bimanual activities were prioritized over detecting activities of daily living, measuring the range of motion, and assessing reaching activities. Regarding activities of daily living, health professionals favored the assessment of eating and dressing compared to other activities. This is in line with family priorities (18, 20), with eating becoming more important for children with severe motor impairments (20).

### Influence of health profession, work experience, and workplace

The opinion of nurses is not sufficiently reflected in the results of this study. Only nine nurses filled out the survey, even though they represent the largest subgroup of health professionals (23). These nine nurses rated the use of walking aids and sports activities as more relevant than other health professions. However, more responses from nurses are needed to draw conclusions about their opinion. Measuring pronation and supination was more relevant for occupational therapists but did not become a top priority in this profession. The relevance rating of the remaining items did not differ between health professions showing a minimal influence of the participants' background on rating the relevance of sensor-based outcomes. An explanation could be that we encouraged the participants to rate the relevance from an interdisciplinary perspective.

In general, work experience in pediatric rehabilitation was positively associated with the relevance rating of sensor-based outcomes, and this correlation was statistically significant for eight survey items. However, the order of the priority list was hardly affected by work experience. For example, measuring the number of sit-to-stand transitions has rank 11 in the overall study population and rank 9 in participants with more than ten years of work experience. Moreover, seven of eight items influenced by work experience showed low priority for experienced and inexperienced health professionals. Hence, we conclude that work experience hardly influences the relevance ratings of outcomes with high priority.

The rating of four items was significantly affected by the workplace. Two of them had low priority anyway, and we argue that the influence of the workplace is negligible for these items. But the distance and speed of walking activities and the number of climbed stairs were more relevant for the staff working at the Swiss Children's Rehab than for those working at other rehabilitation centers. This indicates a higher relevance of gait-related outcomes for our center and should be considered when interpreting and generalizing the results of the current survey.

### Additional outcomes

The sensor-based outcomes of the current survey were derived from previous studies and did not cover the full spectrum of daily motor activities. Besides, these studies were mainly conducted with adult patient populations, and we assumed that child-specific activities were not part of our survey. Therefore, we asked participants after each category and at the end of the survey whether other activities or outcomes would be more relevant for children and adolescents undergoing rehabilitation.

This open-ended question was rarely answered. Still, we want to point out here the most frequently mentioned activities and outcomes: Besides lying, sitting, and standing, which were part of the survey, seven participants mentioned that kneeling and 4-point kneeling should also be considered. This finding suggests that the daily motor activities of adults and children differ and show a need for algorithms discriminating kneeling from other body positions, especially for younger children playing and moving around on the floor. Moreover, eight participants mentioned the relevance of determining the posture of the trunk and pelvis during the different body positions. Determining the gait pattern during walking activities received a relatively high relevance rating compared to other walking-related outcomes. Here, most participants suggested segmenting the gait cycle into stance and swing phases and determining the step length. Another frequently mentioned outcome was the turning speed while patients change their walking direction. However, detecting and quantifying turns in daily life was among the survey items with the lowest relevance rating, and the turning speed was part of this survey item. With regard to joint movements, six participants mentioned the relevance of assessing shoulder flexion and extension, which was not part of the current survey. Hence, it remains unclear if it would have received a higher rating than other joint movements. Still, we expect the measurement of joints' range of motion to be more important on a functional level than in daily life. Other activities mentioned at the end of the survey were playing, crawling, jumping, and communicating. Moreover, participants stated that the required effort and the presence of a supervisor or assistant would be relevant factors when monitoring everyday life motor activities in children and adolescents with neuromotor impairments.

Even though the additional outcomes discussed above were mentioned by multiple participants, their priority with regard to the outcomes of the survey remains unexplored. Hence, future studies are needed to determine the clinical relevance of the additional outcomes mentioned in the open-ended questions of our survey.

### Clinical needs of pediatric rehabilitation to monitor everyday life motor activities

**Figure 3:**
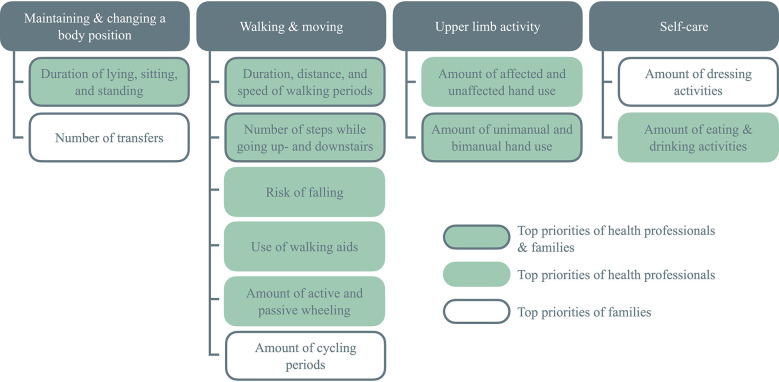
Clinically most relevant outcomes to monitor everyday life motor activities with wearable sensors in children and adolescents with neuromotor impairments.

The results of this survey only reflect the opinion of health professionals and should be complemented with family priorities to provide developers of new algorithms with a comprehensive view on the needs of pediatric rehabilitation. In Figure 3, we have linked the most relevant outcome measures of this survey with the family priorities of our preceding study (18). The summary contains the top ten survey items and the top ten rehabilitation goals which were grouped into four categories: maintaining / changing a body position, walking / moving, upper limb activity, and self-care. We fused the duration, distance, and speed of walking periods to a single outcome measure. Moreover, the rehabilitation goals of walking short and long distances, maintaining a sitting and standing position, as well as putting on and taking of clothes were put together to be in line with the survey items. Eventually, the summary contains the twelve clinically most relevant outcomes to monitor everyday life motor activities.

So far, several sensor systems and algorithms have been developed for a pediatric population. These algorithms cover five of the twelve most relevant outcomes, namely the duration of body positions (8, 9), the amount of active wheeling (10, 11), the hand use laterality and the distinction between unimanual and bimanual activities (13), and the duration, distance, and speed of walking activities (7–10, 12). However, none of these algorithms provide a comprehensive overview of these outcomes and based on the authors' knowledge, the remaining outcomes of the top twelve (i.e., the number of transfers, the risk of falling, the use of walking aids, the number of climbed stairs, and the amount of cycling, dressing, eating and drinking activities) have not been addressed, yet. Consequently, there is a clinical need for new algorithms covering the most important outcomes for children and adolescents with neuromotor impairments.

### Study limitations

The main limitation of this study is the missing definition of the term “clinical relevance” in the survey. Even though all participants were health professionals, their understanding of the term “clinical relevance” could theoretically have ranged from “interesting to know” to “has the potential to change therapy decisions”, which vary considerably in their significance and make the interpretation of the results challenging. Nevertheless, we believe that a clear definition of clinical relevance would have affected the relevance rating of all survey items similarly, rather than their order in the priority list. Besides, the lack of defining clinical relevance also prevented the investigation of the reasons why participants thought sensor-based outcomes would be relevant for pediatric rehabilitation and how they would apply it in clinical practice. Hence, future studies are needed to address these questions.

This study has further limitations. First, the presentation of the sensor-based outcomes might have influenced the relevance rating. If applicable, the survey contained graphical visualizations of fictitious measurements (see Figure 1). This was required to ensure clarity about the meaning of the corresponding survey items. However, the availability and the quality of these visualizations could have resulted in higher priorities for outcome measures with meaningful charts and exemplary data. Second, the interdependency between the survey items' relevance was not investigated in this study (e.g., measuring one outcome might reduce the relevance of measuring other outcomes or two outcomes might only be relevant together). Third, we limited the survey distribution to the German-speaking part of Europe. We chose this approach to cover the opinion of our target population. However, it could affect the generalizability of the study results to other countries. Forth, we expect that mainly technophiles participated in this survey which might be another source of bias. However, we believe this increased the relevance ratings of all survey items similarly and only minimally affected their order in the priority list. Fifth, the response rate of health professionals was not determinable since the number of people who received the link to the online survey is unknown. This could have affected the representativeness of our survey, and based on these five limitations, the relevance ratings of sensor-based outcome measures should be interpreted cautiously. Instead of considering their exact rank in the priority list, their trend of having higher or lower priority for children and adolescents with neuromotor impairments should be acknowledged.

### Conclusion

This survey provides a priority list of sensor-based outcomes to monitor everyday life motor activities of children and adolescents with neuromotor impairments. It reflects the opinion of health professionals and was complemented with the opinion of families of a preceding study to identify the clinical needs of pediatric rehabilitation comprehensively. Knowing these needs will eventually help developers of new algorithms to make the trade-off between deriving clinically meaningful information and minimizing the burden for children and adolescents to wear the sensors in daily life.

## Data Availability

The raw data supporting the conclusions of this article will be made available by the authors, without undue reservation.
