# Supplementary material for: Sensor-based outcomes to monitor everyday life motor activities of children and adolescents with neuromotor impairments: A survey with health professionals
Source: Front Rehabil Sci. 2022 Oct 12;3:865701. doi: 10.3389/fresc.2022.865701 (PMC9596974; doi:10.3389/fresc.2022.865701)
Supplement: Supplementary file 1 [file DataSheet1.pdf]

Everyday life motor activities of children and adolescents

This is an English translation of the survey, which was originally written in German.

Wearable inertial sensors are ideal for monitoring everyday life motor activity. During my doctoral thesis I would like to adapt an existing sensor system to the needs of pediatric rehabilitation. In doing so, I depend on your experience. This survey will give you the opportunity to optimize the sensor system according to your needs and to gain meaningful insight into everyday life motor activities of your patients. For more information about the sensor system, please visit the following website: http://zurichmove.com/

Completing the survey takes about 15 minutes. It can be interrupted at any time and continued later.

Thank you very much for participating in my survey!

Response options

For answering the questions, please imagine that the children and adolescents wear the sensors at home/in their habitual environment which allows for the quantification of their everyday life motor activities (ICF[1] qualifier performance).

On the following pages, possible outcome measures are explained and their results are presented on the basis of a fictitious measurement. Please rate the relevance of these outcome measures for the rehabilitation of children and adolescents. Please answer the questions from an interdisciplinary perspective.

---

[1] International Classification of Functioning, Disability, and Health

Upper limb activity

Upper limb activities can be measured separately for the left and right arm. This enables the quantification of the hand use laterality, the amount of unimanual and bimanual activities, and the diversity of upper limb activities.

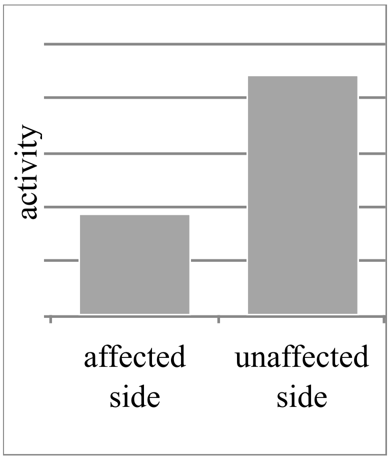

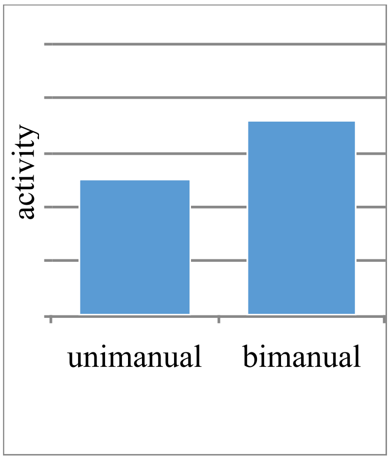

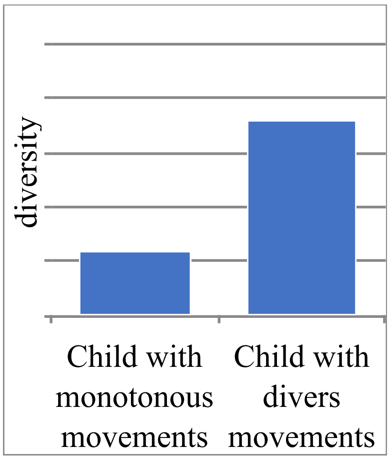

	**very relevant**	**relevant**	**hardly relevant**	**not relevant**	**no answer**
**1. affected vs. unaffected**	○	○	○	○	○
**2. unimanual vs. bimanual**	○	○	○	○	○
**3. diversity**	○	○	○	○	○

If you think other outcome measures would be more relevant in this category, please describe them in the comments box below:

Joint movement in daily life

Joint angles can also be measured. This can be used to quantify the number of repetitions and the range of motion of individual joints in everyday life. Examples from the literature are listed below. The same measurement could be applied to other joints or linked to specific activities (please specify the joints and activities in the comment field if needed).

Fictitious measurement	Supination
# repetitions	335
range of motion	15° ± 3°

	very relevant	relevant	hardly relevant	not relevant	no answer
**4. shoulder ab-/adduction**	○	○	○	○	○
**5. elbow flexion/extension**	○	○	○	○	○
**6. pro-/supination**	○	○	○	○	○
**7. wrist flexion/extension**	○	○	○	○	○
**8. finger flexion/extension**	○	○	○	○	○
**9. knee flexion/extension**	○	○	○	○	○

If you think other outcome measures would be more relevant in this category, please describe them in the comments box below:

Reaching & grasping

Reaching and grasping movements can be detected and evaluated in everyday life. This allows quantifying the number of repetitions as well as the range of reaching forward and sideward relative to the trunk.

Fictitious measurement	Reaching
# repetitions	75
range (forward)	23 cm ± 11 cm
range (sideward)	14 cm ± 4 cm

	very relevant	relevant	hardly relevant	not relevant	no answer
**10. number of repetitions**	◯	◯	◯	◯	◯
**11. range**	◯	◯	◯	◯	◯

If you think other outcome measures would be more relevant in this category, please describe them in the comments box below:

Body positions

Lying, sitting and standing can be recognized in everyday life and the duration a child spends in these body positions is measured. Lying can be subclassified as prone, supine, and side lying, and standing as upright, bending forward, or bending sideward. Other subclassifications or body positions could also be assessed (please specify in the comments field if needed).

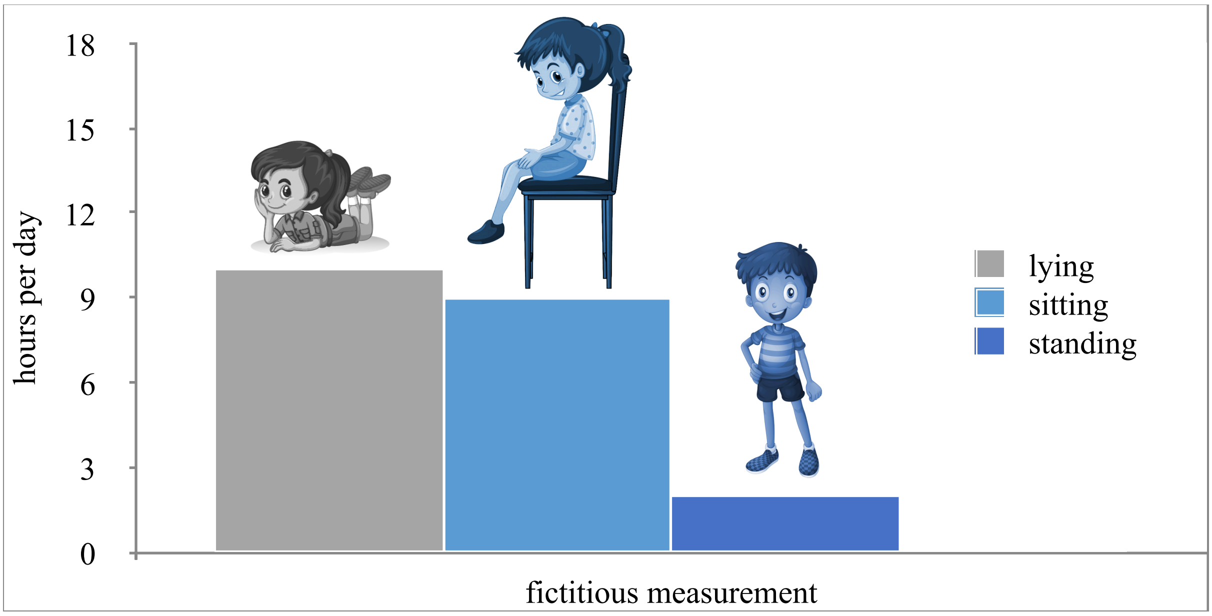

	very relevant	relevant	hardly relevant	not relevant	no answer
**12. lying, sitting, and standing**	◯	◯	◯	◯	◯
**13. lying: prone, supine, etc.**	◯	◯	◯	◯	◯
**14. standing: upright, bent forward, etc.**	◯	◯	◯	◯	◯

If you think other outcome measures would be more relevant in this category, please describe them in the comments box below:

Changing a body position

Transitions between sitting and standing can be detected in everyday life. Then, the quantity (e.g. number of repetitions or duration) or also the quality (e.g. forward tilt of the upper body or flow of movement) can be determined. Transitions between other body positions could also be assessed (please specify in the comments field if needed).

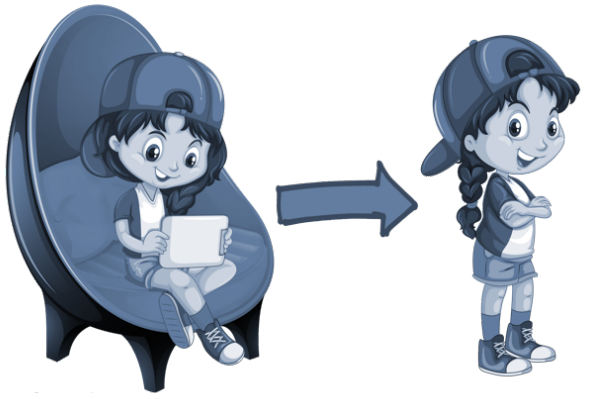

Quantity	Quality
Repetitions:<br>14#	Forward tilt:<br>23° ± 5°
Duration:<br>1.5 s ± 0.6 s	Flow of movement:<br>34 m/s^3 ± 7 m/s^3

	very relevant	relevant	hardly relevant	not relevant	no answer
**15. quantity: sitting <-> standing**	○	○	○	○	○
**16. quality: sitting <-> standing**	○	○	○	○	○

If you think other outcome measures would be more relevant in this category, please describe them in the comments box below:

Walking activity

Walking can be distinguished from other activities, and the daily walking activity can be divided into individual walking bouts. Then, the duration, distance and speed of these bouts can be determined.

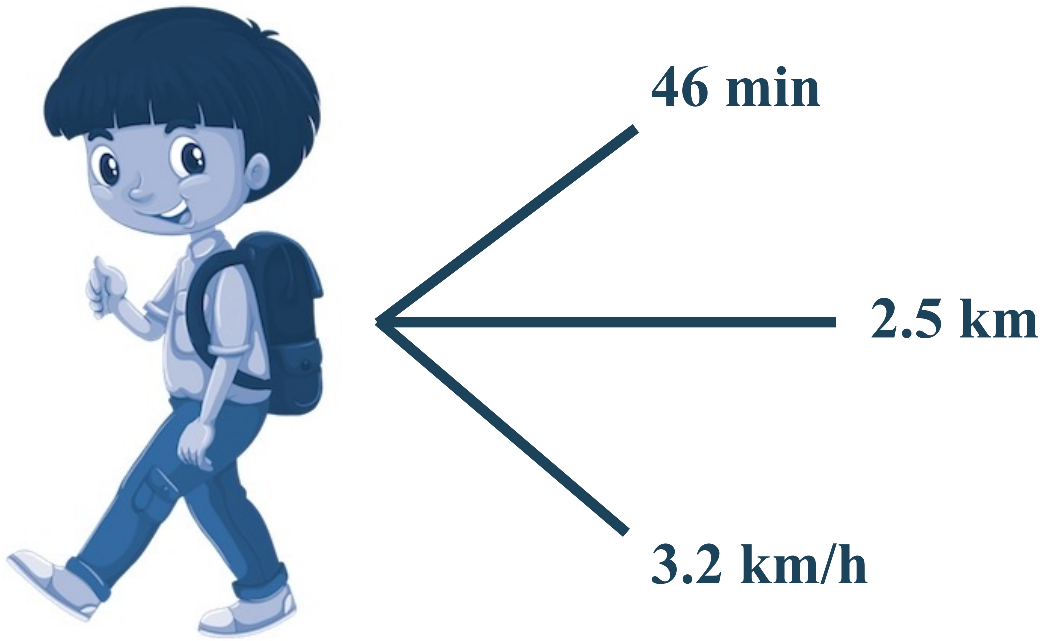

	very relevant	relevant	hardly relevant	not relevant	no answer
**17. duration**	◯	◯	◯	◯	◯
**18. distance, speed**	◯	◯	◯	◯	◯

If you think other outcome measures would be more relevant in this category, please describe them in the comments box below:

Gait parameters

Walking can be segmented into gait cycles which allows quantifying gait parameters such as step length, duration of the stance phase or step symmetry. Please describe the gait parameters in the comment field which are particularly relevant for the rehabilitation of children and adolescents.

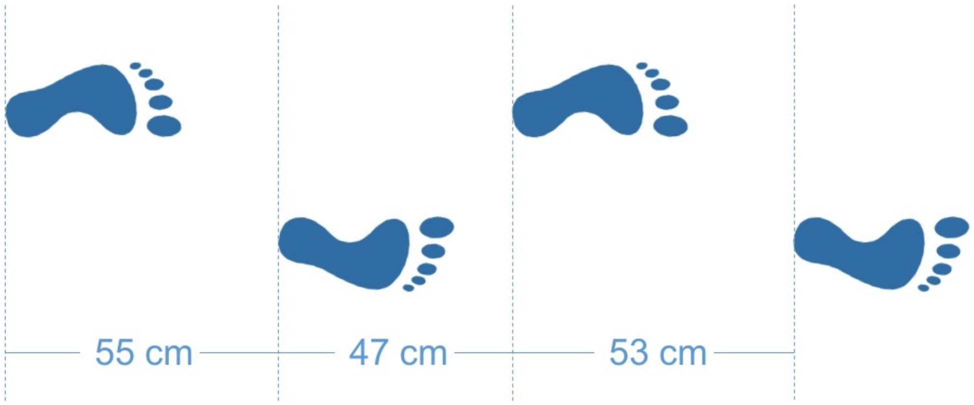

	very relevant	relevant	hardly relevant	not relevant	no answer
**19. gait parameters**	◯	◯	◯	◯	◯

If you think other outcome measures would be more relevant in this category, please describe them in the comments box below:

Walking (risk of falling)

From walking activities, different measures can be calculated that predict a child's risk of falling.

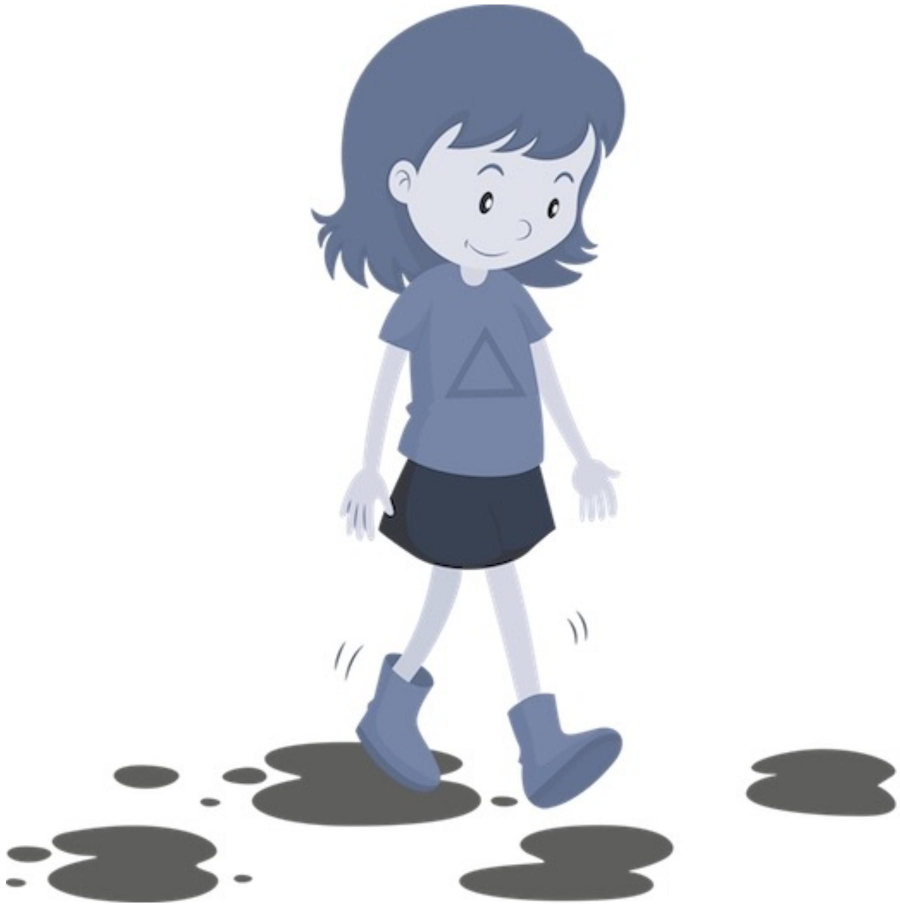

	very relevant	relevant	hardly relevant	not relevant	no answer
**20. risk of falling**	◯	◯	◯	◯	◯

If you think other outcome measures would be more relevant in this category, please describe them in the comments box below:

Walking (turning)

Obstacles or a side road can force a change of direction during walking activities. These turns can be analyzed regarding speed, angular change, number of steps, etc. Please describe the outcome measures in the comment field which are particularly relevant for the rehabilitation of children and adolescents.

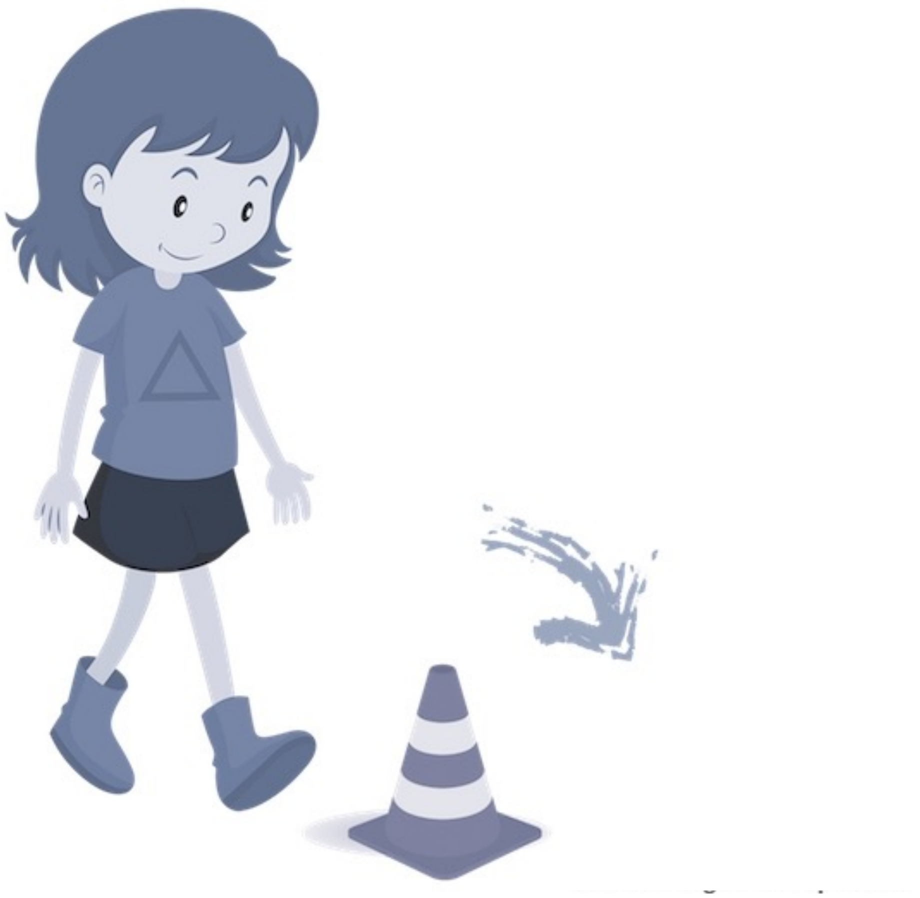

	very relevant	relevant	hardly relevant	not relevant	no answer
**21. walking (turning)**	○	○	○	○	○

If you think other outcome measures would be more relevant in this category, please describe them in the comments box below:

Walking (slope)

The slope of covered walking routes can be measured which allows determining whether a child can walk in steep terrain. In addition, the gait pattern can be compared between level, uphill, and downhill walking.

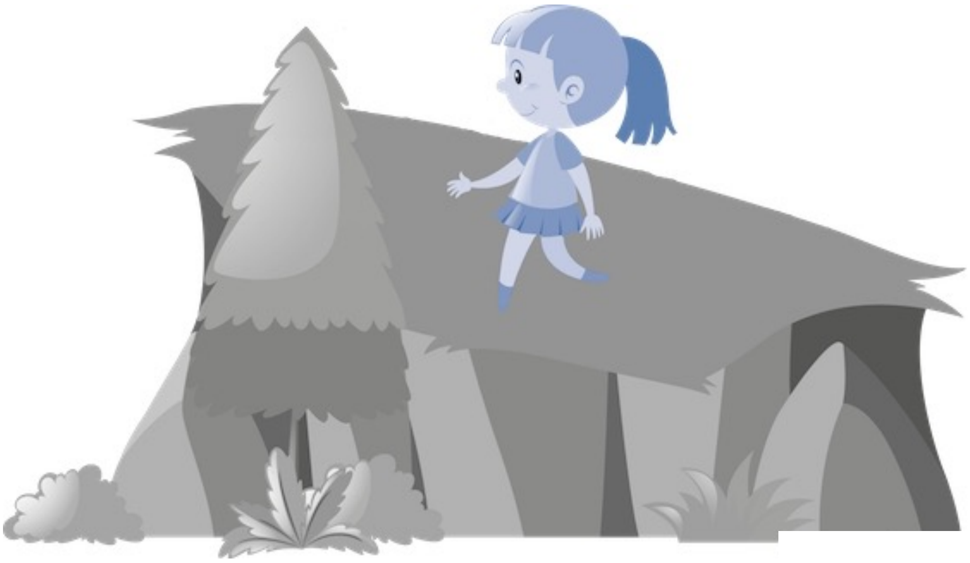

	very relevant	relevant	hardly relevant	not relevant	no answer
**22. walking (slope)**	◯	◯	◯	◯	◯

If you think other outcome measures would be more relevant in this category, please describe them in the comments box below:

Stair climbing

Stair climbing periods and the covered number of steps can be recorded in everyday life (quantity). Furthermore, it can be distinguished between a step-by-step and a step-over-step pattern (quality).

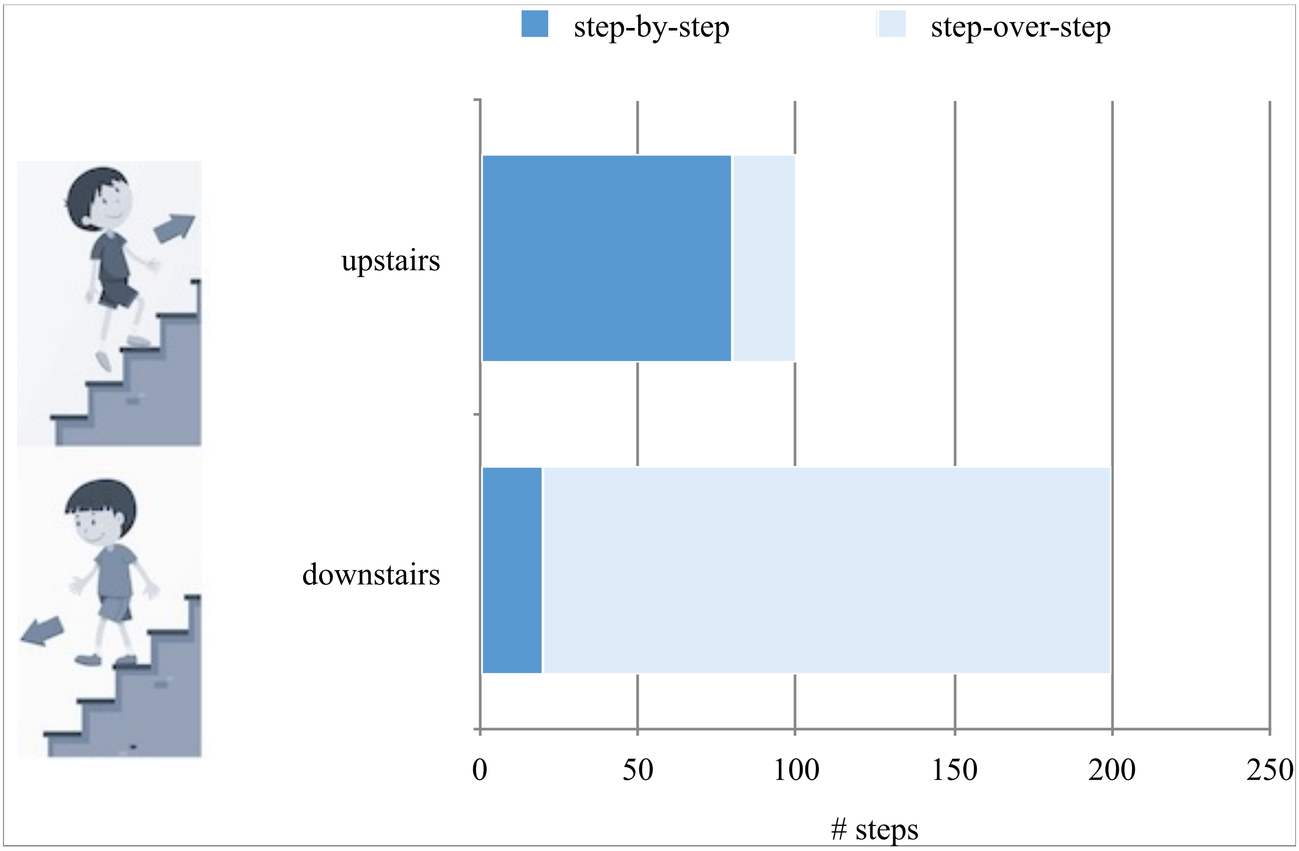

	very relevant	relevant	hardly relevant	not relevant	no answer
**23. number of steps**	○	○	○	○	○
**24. step-by-step vs. step-over-step pattern**	○	○	○	○	○

If you think other outcome measures would be more relevant in this category, please describe them in the comments box below:

Use of walking aids

The use or non-use of assistive devices can be assessed for walking and other activities. Other measures, such as weight bearing or the orientation/position of the assistive device could be determined, too. (please describe them in the comment field if needed).

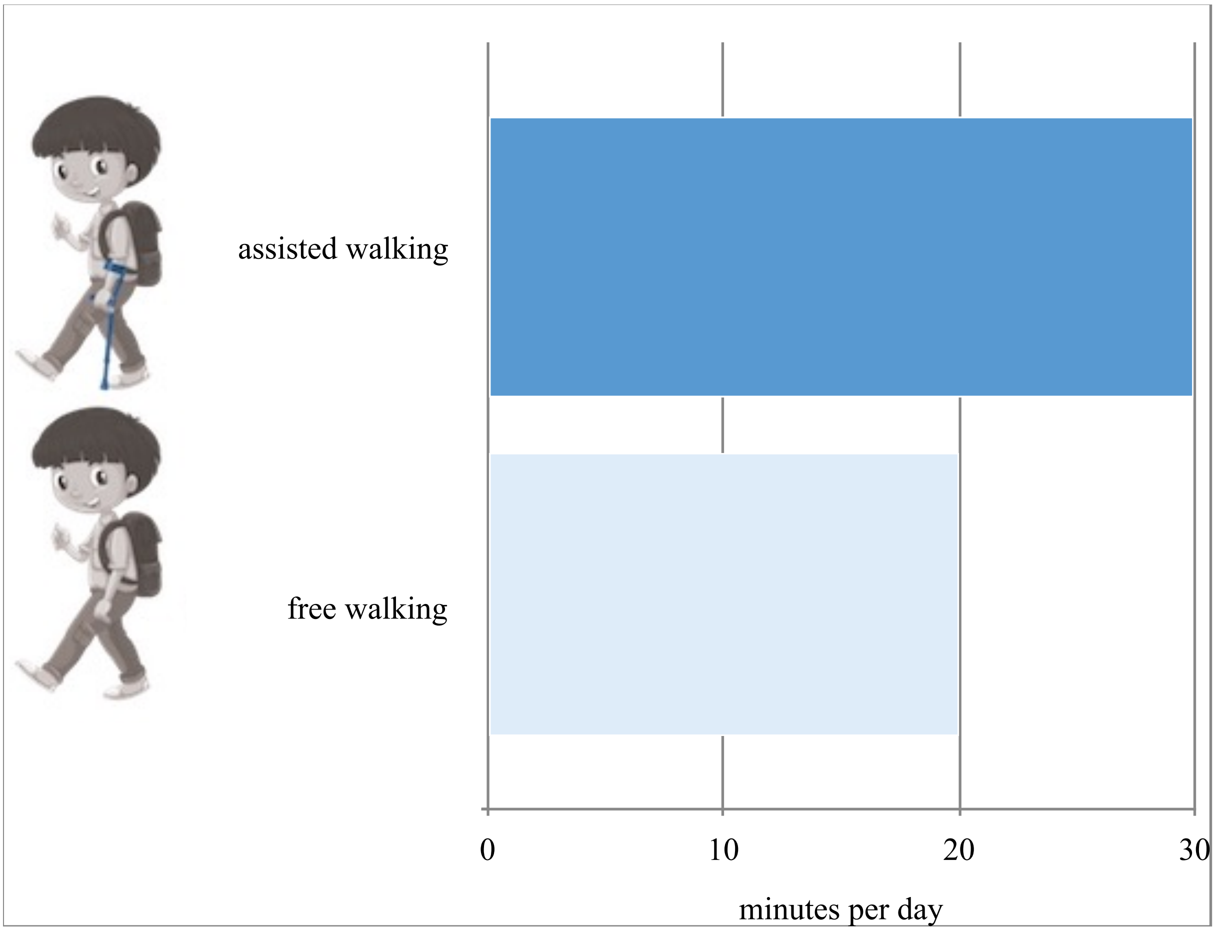

	very relevant	relevant	hardly relevant	not relevant	no answer
**25. Use of aids during walking activities**	○	○	○	○	○

If you think other outcome measures would be more relevant in this category, please describe them in the comments box below:

Wheelchair

Wheeling activities can be detected and subclassified as passive wheeling (being pushed by a third person or a motor) or active self-propulsion. Furthermore, the covered distance and the speed can be determined. Other outcome measures such as the frequency of active strokes or the maneuvering of the wheelchair would also be determined (please describe them in the comment field if needed).

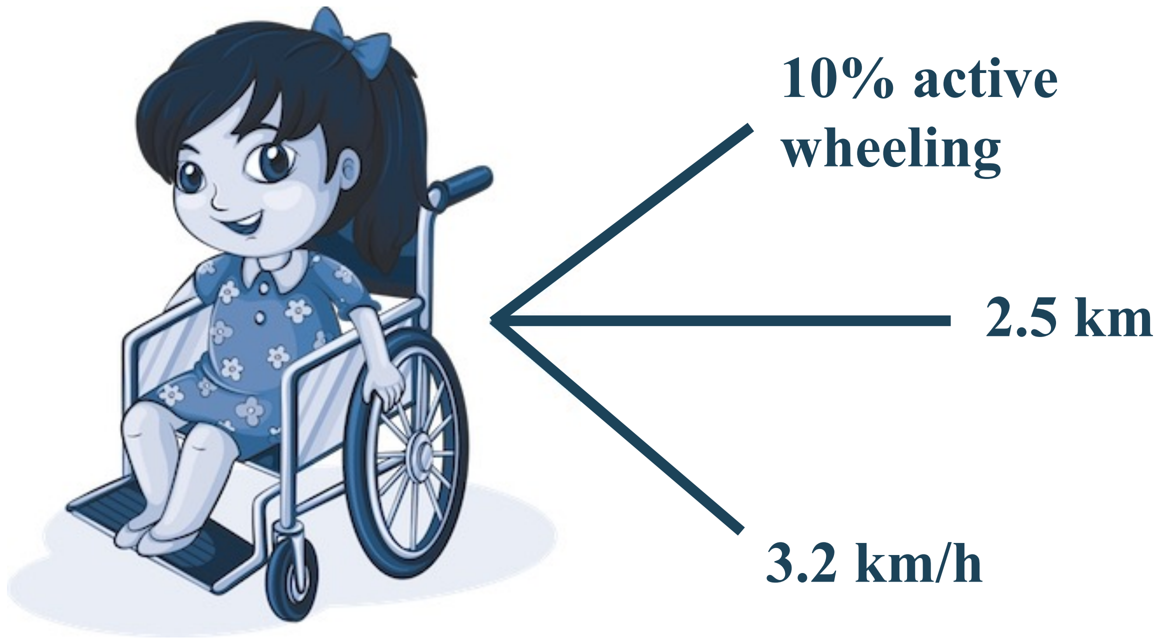

	very relevant	relevant	hardly relevant	not relevant	no answer
**26. active vs. passive**	◯	◯	◯	◯	◯
**27. distance, speed**	◯	◯	◯	◯	◯

If you think other outcome measures would be more relevant in this category, please describe them in the comments box below:

Activities of daily living

Various other activities of daily living can be detected, and the duration or the number of repetitions of these activities can be determined. Here, the activities were grouped because the possibilities are very diverse. If certain activities seem particularly relevant to you, please list them in the comment field.

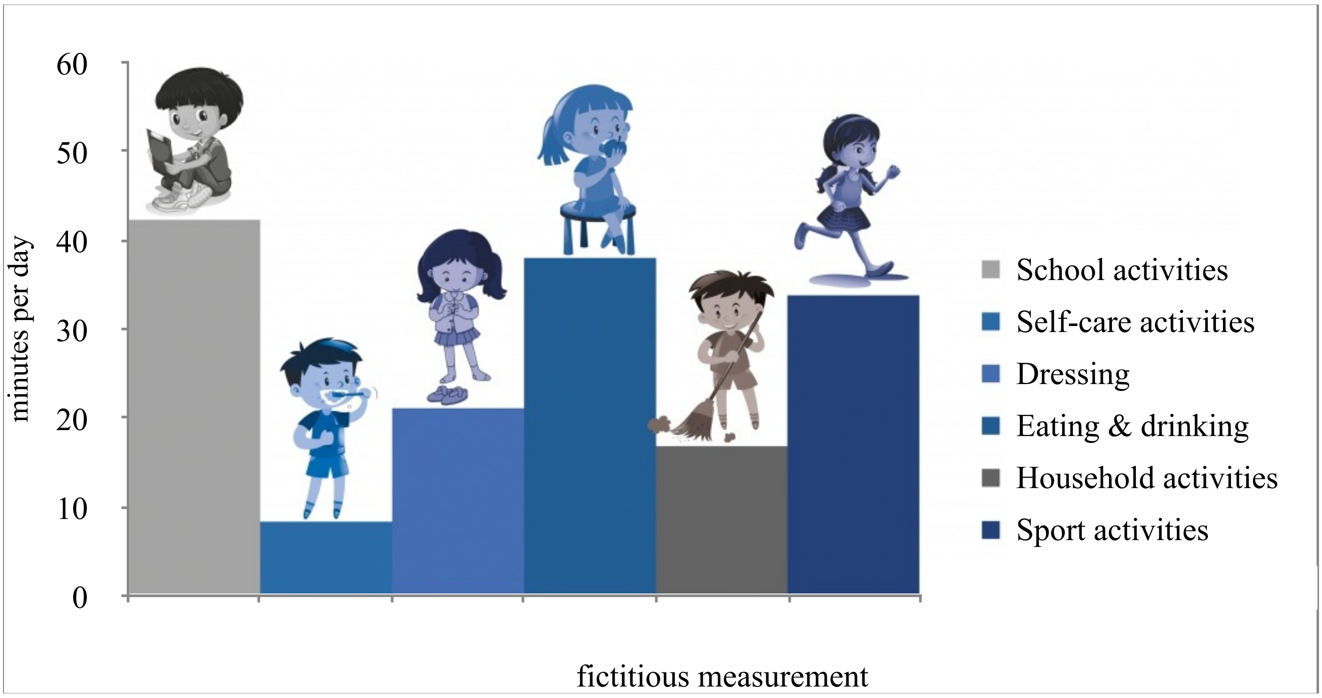

	very relevant	relevant	hardly relevant	not relevant	no answer
**28. school activities (reading, writing)**	○	○	○	○	○
**29. personal hygiene**	○	○	○	○	○
**30. dressing**	○	○	○	○	○
**31. eating & drinking**	○	○	○	○	○
**32. household activities**	○	○	○	○	○
**33. sports activities**	○	○	○	○	○

If you think other outcome measures would be more relevant in this category, please describe them in the comments box below:

Energy expenditure

The intensity of physical activities can be measured and divided into three levels (low, medium and high intensity). This allows determining the daily energy expenditure.

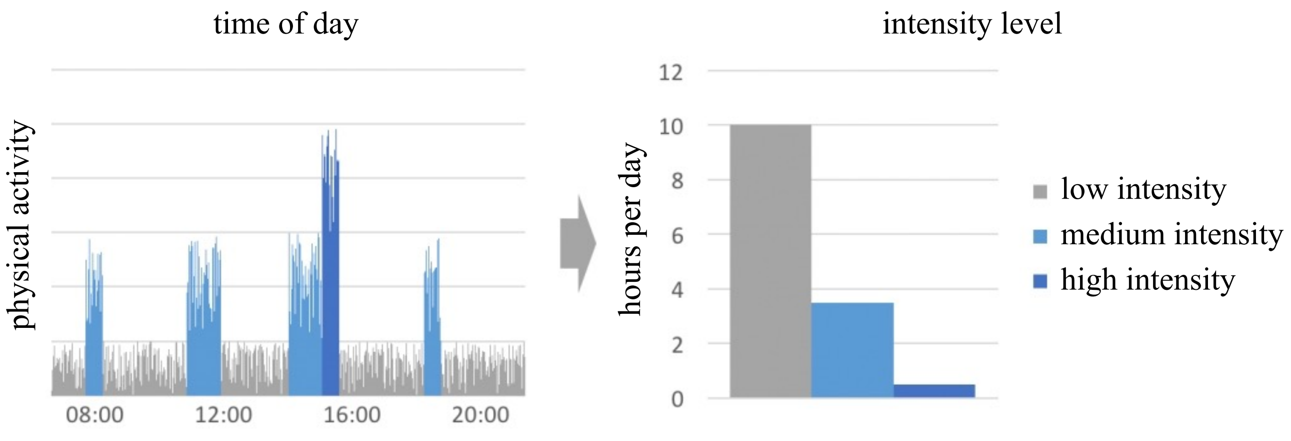

	very relevant	relevant	hardly relevant	not relevant	no answer
**34. energy expenditure**	◯	◯	◯	◯	◯

If you think other outcome measures would be more relevant in this category, please describe them in the comments box below:

What's missing?

The outcome measures in this survey were derived from previous research projects and do not cover all possibilities. If you missed a relevant outcome for the assessment of everyday life motor activities in children and adolescents when filling out this survey, please describe it in the comment field below:

Imagine there would be a sensor system available in the future that derives the outcome measures of this survey. Would you use it to monitor everyday life motor activities?

- ◯ Yes
- ◯ No

Demographic data

Your age (years): ____

Your gender :

- ◯ female
- ◯ male

Profession:

- ◯ doctor
- ◯ movement scientist
- ◯ occupational therapist
- ◯ physiotherapist
- ◯ nurse
- ◯ sport therapist
- ◯ other: _________

Work experience in pediatrics (years): ____

Workplace: ____________________

Credits

The illustrations of this survey were designed using resources from freepik.com
